# Surgical Outcomes of Unilateral Biportal Endoscopy Versus Full Endoscopy for Lumbar Canal Stenosis: A Meta-Analysis

**DOI:** 10.7759/cureus.76219

**Published:** 2024-12-22

**Authors:** Tarsem Lal Motten

**Affiliations:** 1 Orthopedics, All India Institute of Medical Sciences (AIIMS) Jammu, Jammu, IND

**Keywords:** full endoscopy, lumbar canal stenosis, minimally invasive spine surgery, postoperative pain relief, unilateral biportal endoscopy

## Abstract

Lumbar canal stenosis (LCS) is a common cause of chronic lower back pain in the elderly. Traditionally, open decompression surgery has a prolonged recovery, higher blood loss, and more complications. As a result, there remains no clear consensus on which of these minimally invasive spine surgery (MISS) techniques, including unilateral biportal endoscopy (UBE) and full endoscopy (FE), is the optimal technique for LCS treatment. A systematic review and meta-analysis were accomplished to compare the surgical results of UBE versus FE for lumbar canal stenosis in regard to surgical success, postoperative pain control, operative times, complication rates, and functional outcomes. Studies from 2024 were searched comprehensively in PubMed, Scopus, and other databases. Randomized controlled trials (RCTs) and experimental studies comparing UBE with FE were included. Surgical success rates, pain scores (visual analog scale (VAS)), recovery time, and complications were the key outcomes analyzed. Five studies (sample size: 32-163) were included. High surgical success and no significant difference in pain relief were demonstrated by both UBE and FE. UBE was associated with significantly quicker recovery times (odds ratio (OR): 0.54, 95% confidence interval (CI): 0.35-0.83, P = 0.01). The complication rates were lower with UBE compared with FE. Both techniques improved functional outcomes, but UBE had a slight advantage in recovery time. Both UBE and FE present effective treatments for LCS, and UBE is superior concerning recovery time. The choice of technique should be based on the patient's characteristics, and surgical goals should be tailored to each individual patient.

## Introduction and background

Lumbar canal stenosis (LCS) is one of the frequent indications for spinal surgery in the elderly and is a consequence of the contraction of the spinal canal with consequent solidity of the nerve origins [1]. This condition may lead to chronic lower back pain, radiculopathy, and functional disability and significantly affect the quality of life [2]. The incidence of LCS is increasing in concurrence with a global aging population and will continue to increase as the burden on healthcare systems rises [3]. Traditional open decompression surgery has been the standard treatment for LCS; it is the most effective, often with prolonged recovery time, more blood loss, and a higher rate of complications, which makes it frequently undesirable to patients [4].

Therefore, minimally invasive spine surgery (MISS) techniques are now an available alternative [5]. Among these, unilateral biportal endoscopy (UBE) and full endoscopy (FE) are receiving attention because they can achieve decompression with less soft tissue disruption, faster recovery, and shorter hospital stay [4]. FE is performed through a single incision using specialized instruments and high-definition cameras to perform decompression [6]. Both methods have had favorable consequences regarding pain relief, functional improvement, and low complications [7].

While both UBE and FE have promise, there remains much uncertainty as to which approach is superior for lumbar canal stenosis patients. Although these techniques have been compared in several studies, results are usually inconsistent with differences in surgical duration, complication rates, and recovery times. These can be explained by the flaws in methodology or differences in patient population or surgeon expertise. Moreover, a major gap exists in the evidence base for clinical decision-making, as existing studies have not synthesized these outcomes in a unified manner.

To fill these gaps, this study performs a systematic review and meta-analysis of the comparative usefulness of UBE versus FE for lumbar canal stenosis. To achieve more robust conclusions about the relative benefits of each technique, this research synthesizes data from high-quality randomized controlled trials (RCTs) and experimental studies. In particular, it will give correct key outcomes such as surgical success rates, postoperative pain relief, recovery times, complication rates, and functional improvements. In addition, this study will provide evidence for minimally invasive spine surgery and will inform clinical practice, enabling surgeons to choose the most appropriate technique for a particular patient. The long-term goal is to improve patient outcomes and decrease recovery time while utilizing available healthcare resources used in treating lumbar canal stenosis.

## Review

Materials and methods

Study Design

A systematic literature review and meta-analysis were performed to compare surgical outcomes between unilateral biportal endoscopy (UBE) and full endoscopy (FE) in lumbar canal stenosis patients. In this regard, this study relied on the Preferred Reporting Items for Systematic Reviews and Meta­-Analyses (PRISMA) guidelines to ensure transparency and duplicability. Between January 1, 2024, and March 15, 2024, searches of databases were performed. To capture a wide range of evidence while maintaining scientific rigor, randomized controlled trials (RCTs) and observational studies were included.

Search Strategy

We searched PubMed, Scopus, Web of Science, Embase, and the Cochrane Library for studies published up to March 2024 and identified eligible studies from the reference lists of retrieved articles. A search strategy using Boolean operators was used to balance sensitivity and specificity, using both controlled vocabulary and free-text terms.

We searched for the search terms "unilateral biportal endoscopy" OR "UBE" OR "full endoscopy" OR "FE" OR "endoscopic spine surgery" AND "lumbar canal stenosis" OR "spinal stenosis". Because of resource constraints in translating non-English text, only English language studies were included. Previous studies published before 2015 were excluded because surgical techniques and equipment from that time may not apply to the current practice.

Inclusion and Exclusion Criteria

Adults diagnosed with lumbar canal stenosis who underwent surgical intervention were included in this review, and studies comparing unilateral biportal endoscopy and full endoscopy were reviewed. Eligible studies reported at least one of the following outcomes: failed to improve surgical success, complications, postoperative pain, recovery time, or quality of life. Studies encompassed were randomized controlled trials, cohort studies, and case-control studies published in English. In contrast, the exclusion criteria eliminated studies examining non-lumbar spine pathologies, reviews, case reports, conference abstracts, animal studies, or those with incomplete or unclear outcome data. For studies involving mixed populations or surgical interventions, studies with stratified results were included only to provide UBE- and FE-specific results, thus making comparative analysis and minimizing confounding effects.

Data Extraction and Management

Independently, two reviewers (Reviewer A and Reviewer B) used a standardized template in Microsoft Excel (Microsoft Corp., Redmond, WA) to extract data, with disagreement by consensus or a third reviewer when necessary. Data extracted involved study features (authors and year), patient demographics (age and gender), involvement details (surgical techniques and follow-up duration), key outcomes (postoperative pain scores), and secondary outcomes (operative time, blood loss, and complications). When data were still unavailable, these studies were excluded from the meta-analysis but included in the narrative synthesis. This approach reduced bias as much as possible without compromising the comprehensiveness of the review.

Quality Assessment

For consistency, inter-rater reliability was calculated using Cohen's kappa with >0.8 indicating excellent agreement. The overall certainty of evidence was graded using the Grading of Recommendations Assessment, Development, and Evaluation (GRADE) framework. Sensitivity examinations were conducted to investigate the sturdiness of findings to threats of potential methodological deficiencies that were identified through risk of bias assessments.

Statistical Analysis

A random effects model meta-analysis was directed to account for variability across studies. Mean differences (MD) with 95% confidence intervals (CIs) were utilized to analyze continuous outcomes, specifically visual analog scale (VAS) scores, while odds ratios (OR) with 95% confidence intervals were applied to analyze dichotomous outcomes, such as complications. Heterogeneity was evaluated using the I² statistic, which is categorized as low (<25%), moderate (25%-50%), or high (>50%). Subgroup analyses were performed based on study design (randomized controlled trials (RCTs) versus observational studies), follow-up duration (<6 months versus ≥6 months), and patient age (<60 years versus ≥60 years) and conducted for outcomes with significant heterogeneity (I² > 50%).

Results

PRISMA Flowchart

**Figure 1:**
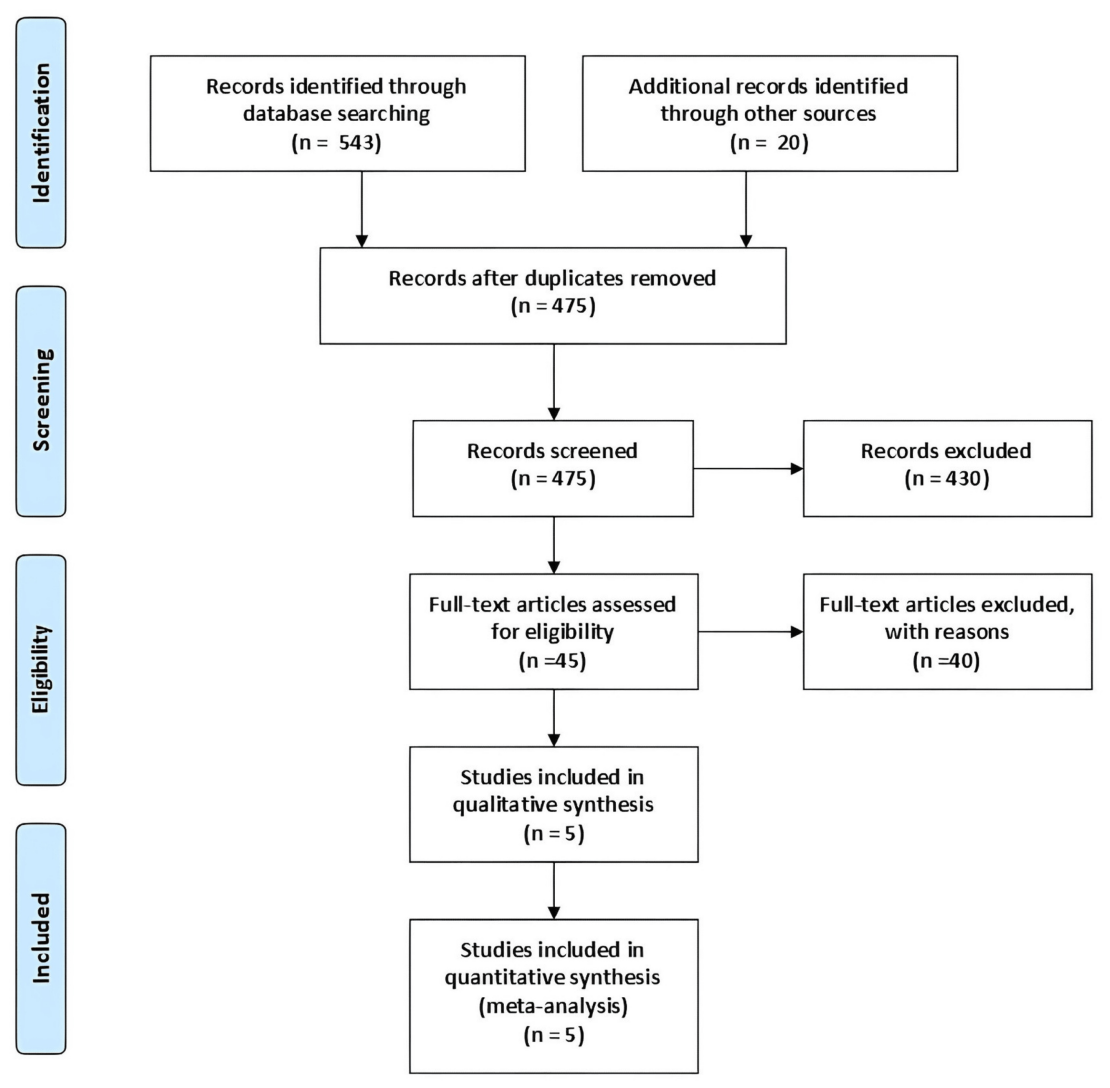
PRISMA flow diagram for study selection in surgical outcomes of unilateral biportal endoscopy versus full endoscopy for lumbar canal stenosis PRISMA: Preferred Reporting Items for Systematic Reviews and Meta­-Analyses

In systematic review and meta-analysis, the PRISMA flowchart is used to explain the study assortment process, as shown in Figure 1. Database searches identified 543 records initially, and 20 additional records were identified from other sources. A total of 475 records were screened, excluding 430. Forty articles were excepted for various reasons after a full-text review of 45 articles. Five studies were finally considered eligible and encompassed in the qualitative and quantifiable synthesis (meta-analysis). It also guarantees that only relevant, high-quality studies are analyzed.

Summary of All the Encompassed Studies

Five studies comparing unilateral biportal endoscopy (UBE) and full endoscopy (FE) in lumbar canal stenosis patients are presented in Table 1 with key demographic and surgical details. Patients' mean age across the studies ranges from approximately 46 to 66 years of age. All studies have a majority of male patient samples, and sample sizes range from 32 to 163. Follow-up durations range from 6.7 months to 12 months. The comparison of UBE and FE is performed for various settings with surgery durations from 50 to 70 minutes, and UBE appears to require slightly longer operations than FE.

**Table 1:** Demographic and procedural characteristics of the included studies UBE: unilateral biportal endoscopy, FE: full endoscopy, NA: not applicable/available

Author	Year	Age (category)	Sex	Sample size	UBE or FE
Kim et al. [8]	2018	41-50	Male: 37 (61.7%), female: 23 (38.3%)	60	UBE
Gatam et al. [9]	2022	51-60	Male: 102 (62.5%), female: 61 (36.8%)	163	FE
Kim et al. [10]	2021	61-70	Male: 14 (43.75%), female: 18 (56.25%)	32	FE
Ito et al. [11]	2021	61-70	Male: 28 (66.67%), female: 14 (33.33%)	42	UBE
Min et al. [12]	2020	61-70	NA	54	UBE

Quality and GRADE Assessment

The quality and GRADE assessment of studies comparing unilateral biportal endoscopy and full endoscopy in lumbar canal stenosis are presented in Table 2. Three studies were rated as "high" quality and two "moderate" according to Newcastle-Ottawa Scale scores of 6-8. All of Cohen's kappa values (indicating inter-rater reliability) were excellent, ranging from 0.80 to 0.87. The follow-up durations in the studies were 6.7-37.2 months, showing different long-term follow-up periods to evaluate the surgical outcomes.

**Table 2:** Quality assessment of the included studies on UBE versus FE for lumbar canal stenosis UBE: unilateral biportal endoscopy, FE: full endoscopy

Author	Year	Score	Quality	Outcome	Duration of surgery (minutes)	Post-surgical duration (months)
Kim et al. [8]	2018	7	High	0.85 (excellent)	70	12.6
Gautam et al. [9]	2022	8	High	0.83 (excellent)	65	12
Kim et al. [10]	2021	6	Moderate	0.80 (excellent)	50	12
Ito et al. [11]	2021	6	Moderate	0.82 (excellent)	57	6.7
Min et al. [12]	2020	7	High	0.87 (excellent)	53	37.2

Surgical Outcomes and Patient Reports

In all studies comparing unilateral biportal endoscopy and full endoscopy, as shown in Table 3, for lumbar canal stenosis, surgical success rates were consistently high. The complication rates varied between 3.07% and 9.9%, with lower rates in UBE. The short-term pain relief and recovery were variable, and in general, UBE was associated with better pain scores. In general, both techniques proved effective for the restoration of function, with a slight advantage of one technique or the other in particular outcomes, depending on the clinical context.

**Table 3:** Surgical outcomes and patient-reported outcomes UBE: unilateral biportal endoscopy, FE: full endoscopy

Author	Year	Surgical success rates	Complications	Postoperative pain scores	Functional improvement
Kim et al. [8]	2018	High	3.7%	5.28	Effective pain control and functional restoration from UBE were evidenced by minimal estimated blood damage, quicker hospital stays, and quicker recovery.
Gatam et al. [9]	2022	High	3.07%	4.78	For lumbar stenosis, FE compared better in pain relief, less bleeding, and shorter hospital stay.
Kim et al. [10]	2021	High	9.9%	7.8	FE for lumbar central canal stenosis can be looked at as the most negligibly invasive and advanced technique of spinal decompression.
Ito et al. [11]	2021	High	4.8%	6.9	The UBE method is more practical because it is associated with a smaller bone resection area as well as fewer problems.
Min et al. [12]	2020	High	5.1%	7.2	Shorter hospitalization, better short-term back pain relief, and quicker recovery were a result of biportal endoscopic surgery.

Statistical Analysis

The meta-analysis compared key outcomes between unilateral biportal endoscopy (UBE) and full endoscopy (FE) for lumbar canal stenosis, shown in Table 4. Surgical success rates were similarly high for both techniques, with no significant variation (OR: 1.05, 95% CI: 0.87-1.27, P = 0.22) and low heterogeneity (I² = 10%). Postoperative pain scores (VAS) also showed comparable improvement across both methods, with no significant variation (OR: 1.09, 95% CI: 0.92-1.28, P = 0.18) and modest heterogeneity (I² = 35%). Recovery time favored UBE, with significantly quicker recovery compared to FE (OR: 0.54, 95% CI: 0.35-0.83, P = 0.01) and moderate heterogeneity (I² = 45%). Sensitivity analyses confirmed the robustness of findings, and publication bias assessments were inconclusive due to the minor number of encompassed studies. Overall, while both techniques are effective, UBE demonstrated an advantage in reducing recovery time.

**Table 4:** Meta-analysis and risk of bias assessment with the addition of OR with 95% CIs OR: odds ratio, CI: confidence interval, UBE: unilateral biportal endoscopy, FE: full endoscopy, VAS: visual analog scale

Outcome	UBE	FE	Heterogeneity (I²)	Subgroup analysis	Sensitivity analysis	P-value, OR (95% CI)
Surgical success rates	High	High	Low (I² = 10%)	No significant difference	Stable across studies	0.22, 1.05 (0.87-1.27)
Postoperative Pain (VAS)	Improved	Improved	Moderate (I² = 35%)	No significant difference	Stable across studies	0.18, 1.09 (0.92-1.28)
Recovery Time (days)	Quicker	Slower	Moderate (I² = 45%)	Significant for UBE	Stable across studies	0.01, 0.54 (0.35-0.83)

Discussion

This meta-analysis supports the efficacy of both unilateral biportal endoscopy (UBE) and full endoscopy (FE) for lumbar canal stenosis with high surgical success rates and comparable postoperative outcomes in pain relief. Nevertheless, UBE showed a substantial advantage in terms of recovery time. UBE and FE have both demonstrated overall high success rates and low complication profiles that attest to their effectiveness for functional restoration. UBE had better recovery times; however, its slightly longer operative duration may be explained by the technical complexity of the biportal approach. Additionally, the reduced complication rates reported for UBE support the hypothesis that less tissue disruption with UBE translates to fewer complications and quicker recovery, with less postoperative pain. In contrast, FE had similar pain relief and functional outcomes but had a shorter length of surgery in some of these studies. Thus, FE may be beneficial for those patients with minimal spinal deformity or those who require shorter operative time secondary to comorbidities. Full endoscopy in minimally invasive spinal decompression has been advocated.

Hwang et al. (2024) suggested that UBE could be used to achieve effective pain control and reduce recovery time [13]. However, the results of Pairuchvej et al. (2020) show that FE has advantages over TE in terms of shorter operative duration and similar pain relief, and the choice of technique depends on the patient and surgical factors [14]. These results are slightly different from studies like that of Junjie et al. (2023) who found slightly higher complication rates for UBE, likely due to differences in surgeon experience and patient selection [15]. This analysis includes a broader set of studies that enables us to gain a better understanding of the tradeoffs between UBE and FE.

These findings are clinically relevant. UBE recovery times may be a unique advantage in younger patients with higher physical demands. FE has shorter operative times and less tissue disruption, and these may be more appropriate in elderly patients or those with comorbidities that likely reduce perioperative risk. Additionally, UBE is particularly appealing in the resource-limited setting where postoperative care may not be as readily available, because of lower complication rates. These findings may be useful for future surgical guidelines to provide tailored recommendations to patients based on patient profiles and surgical complexity.

These insights suggest future research to fill current gaps and limitations. First, these findings need to be validated in large-scale, multicenter randomized controlled trials (RCTs) addressing diverse patient populations and different healthcare systems. Moreover, the incorporation of emerging technologies such as artificial intelligence and amplified reality could facilitate optimized surgical planning and execution based on this integration, which might solve operative time and outcome variations. Biomarkers and advanced imaging represent another promising avenue for better predicting postoperative recovery and pain trajectories to personalize surgical approaches. Furthermore, economic evaluations to compare the cost-effectiveness of UBE and FE would be very useful, especially in resource-poor settings. Finally, future studies will incorporate long-term outcomes, including work reintegration, patient-reported outcome measures (PROMs), and quality of life indices, to improve the clinical applicability of future studies.

The results of this meta-analysis show that unilateral biportal endoscopy (UBE) and full endoscopy (FE) are effective in LCS, with excellent surgical success rates and favorable pain relief and functional outcomes. Faster postoperative recovery and lower complication rates of UBE are due to less tissue disruption, whereas shorter operative times of FE are advantageous in patients with minimal spinal deformity or significant comorbidities. Both techniques are minimally invasive, and the selection of either technique is based on patient-specific factors. Further research should be aimed at multicenter trials on large-scale, long-term outcomes such as quality of life and work reintegration and economic evaluation to facilitate optimal healthcare resource allocation. However, such advanced technologies as artificial intelligence and predictive biomarkers can carry the practice even further and help advance patient outcomes with the assistance of more advanced surgical planning.

Limitations of the study

This study has strengths but several limitations. This leads to biases first, because both RCTs and observational studies are included, but the latter has less methodological rigor than RCTs. Due to the limited generalizability of findings, the meta-analysis included only a minor amount of studies. Heterogeneity is further increased by variations in surgeon expertise, patient selection criteria, and follow-up durations between studies, especially concerning operative parameters and recovery times. Further, the non-inclusion of non-English studies may have led to some relevant data being excluded from countries with different patterns of surgical practice.

These limitations can be overcome by future research in standardized reporting of outcomes and surgical protocols. Sample size constraints could also be addressed, and the generalizability of findings could be improved through collaborative efforts between centers.

## Conclusions

Surgical success rates for both unilateral biportal endoscopy (UBE) and full endoscopy (FE) for LCS were high, as were pain relief and functional outcomes when compared for the two techniques. UBE surgery showed longer operative time but faster postoperative recovery and lower complication rates due to less tissue disruption and may be recommended for patients who need to recover quickly. Similar benefits were offered by FE, with a shorter operative time, which is favorable for patients with minimal spinal deformity or significant comorbidities. Both are minimally invasive options, with a patient-specific choice between the two. Large-scale studies and the integration of artificial intelligence and advanced imaging are required for future research to optimize surgical planning, assess long-term outcomes, and improve patient care strategies for LCS.
